# Residual Human Intestinal Nematode Infections Following Discontinuation of Mass Drug Administration in a Rural South Asian Setting: Implications for Deworming Surveillance

**DOI:** 10.3390/tropicalmed11060147

**Published:** 2026-05-27

**Authors:** Nalini Jayakody, Catherine A. Gordon, Anjana Silva, Nuwan Wickramasinghe, Susiji Wickramasinghe, Natasha Collinson, Asela Wijayasekara, Chanaka Karunarathne, Nilanthi de Silva, Kosala Weerakoon

**Affiliations:** 1 Department of Parasitology, Faculty of Medicine, Wayamba University of Sri Lanka, Kuliyapitiya 60200, Sri Lanka; asela@wyb.ac.lk; 2 Department of Parasitology, Faculty of Medicine and Allied Sciences, Rajarata University of Sri Lanka, Saliyapura 50008, Sri Lanka; 3 Infection and Inflammation Program, Applied Tropical and Molecular Parasitology Laboratory, QIMR Berghofer Medical Research Institute, Brisbane 4006, Queensland, Australia; 4 Faculty of Medicine, The University of Queensland, Brisbane 4072, Queensland, Australia; 5 Center for Tropical Health and Emerging Diseases, QIMR Berghofer Medical Research Institute, Brisbane 4006, Queensland, Australia; 6 Department of Community Medicine, Faculty of Medicine and Allied Sciences, Rajarata University of Sri Lanka, Saliyapura 50008, Sri Lanka; 7 Department of Parasitology, Faculty of Medicine, University of Peradeniya, Kandy 20400, Sri Lanka; 8 Department of Physiology, Faculty of Medicine, Wayamba University of Sri Lanka, Kuliyapitiya 60200, Sri Lanka; chanaka85@wyb.ac.lk; 9 Department of Parasitology, Faculty of Medicine, University of Kelaniya, Ragama 11010, Sri Lanka

**Keywords:** human intestinal nematode infections, mass drug administration, microscopy, polymerase chain reaction, qPCR, soil-transmitted helminths, strongyloidiasis, Sri Lanka

## Abstract

Prevalence of soil-transmitted helminth (STH) infections in Sri Lanka has declined from 73.3% in 1954 to <1% in 2017, leading to revised deworming policies and discontinuation of routine deworming in three districts, including Anuradhapura, in 2019. As countries approach elimination, this study assessed human intestinal nematode infection (HINI) prevalence in Anuradhapura, a low-endemic setting, using microscopic and molecular methods. From January to November 2023, 967 primary school children were recruited and screened for HINI using direct smears, Kato–Katz, formalin ether concentration, agar plate culture, scotch tape, and qPCR. Combined microscopy and molecular prevalence was 41.2% for STH and 54.1% for HINI. The most prevalent HINI was *Enterobius vermicularis* (191, 24.5%), followed by *Strongyloides stercoralis* (120, 19.2%), *Ascaris lumbricoides* (113, 18.1%), *Trichuris trichiura* (40, 6.4%), and hookworm (43, 6.9%). Against the composite reference standard, qPCR demonstrated superior diagnostic performance across all species, achieving sensitivities of 86.7% for *Ascaris*, 85.0% for *Trichuris*, and 97.7% for hookworm, consistently exceeding those of microscopy-based methods. Correspondingly, qPCR also identified the highest infection prevalences, detecting *Ascaris*, *Trichuris*, and hookworm infections in 15.7%, 5.4%, and 6.7% of participants, respectively. All infections were low-intensity as determined by Kato–Katz. Microscopy significantly underestimates STH prevalence. In post-deworming settings, integrated surveillance including molecular diagnostics is essential. The high prevalence of *S. stercoralis* and *E. vermicularis* highlights the need for expanded surveillance and targeted interventions to support sustainable control.

## 1. Introduction

Human intestinal nematode infections (HINIs) remain among the most prevalent neglected tropical diseases (NTDs) globally, affecting an estimated 1.5 billion people and contributing substantially to morbidity, particularly in low and middle-income countries [1]. The primary species include *Ascaris lumbricoides*, *Trichuris trichiura*, hookworms (*Necator americanus*, *Ancylostoma duodenale*), *Strongyloides stercoralis*, and *Enterobius vermicularis* [2]. Transmission occurs through ingestion of infective eggs or skin penetration by larvae, facilitated by inadequate water, sanitation, and hygiene (WASH) conditions and healthcare access [3]. High-risk groups include children, pregnant women, immunocompromised individuals, and those with occupational soil exposure, particularly in settings with low deworming coverage [4]. Although often asymptomatic, chronic infections can lead to malnutrition, anaemia, and impaired physical and cognitive development, particularly in children [5].

Among HINIs, soil-transmitted helminths (STHs), comprising *A. lumbricoides*, *T. trichiura*, hookworms and *S. stercoralis*, represent a major subgroup defined by their reliance on soil for the development of infective stages [6]. This environmental transmission pathway makes STHs particularly amenable to large-scale control through preventive chemotherapy. Accordingly, global control efforts have focused on mass drug administration (MDA), leading to substantial reductions in STH prevalence in many endemic settings [1,3]. However, as countries transition from morbidity control to elimination-oriented strategies, a key challenge is the detection of low-intensity infections that allow residual transmission. Conventional microscopy, particularly Kato–Katz (KK), remains the cornerstone of surveillance but has reduced sensitivity in low-prevalence settings, potentially underestimating the true infection burden [7]. This limitation is particularly important in post-MDA settings, where infections are often of low intensity and intermittently shed, reducing the likelihood of microscopic detection. Molecular methods such as quantitative polymerase chain reaction (qPCR) provide substantially higher analytical sensitivity and species-specific detection, enabling identification of low-burden and otherwise undetected infections [8,9,10].

Sri Lanka represents a model setting for this transition. Following over a century of documented transmission and decades of MDA alongside improvements in WASH, national STH prevalence declined to 0.97% by 2017 [11,12,13,14]. This latest estimate, derived from a national survey among primary school children (PSC) aged 5–7 years using the modified duplicate KK test, guided the revision of national deworming guidelines in 2018. As a result, regular deworming was discontinued in low-prevalence districts, including Anuradhapura (0.21%), in line with WHO recommendations [15,16]. However, prior national surveys relied solely on microscopy and did not assess *S. stercralis* or *E. vermicularis*, raising concerns regarding undetected residual infections. Evidence from other endemic regions demonstrates that molecular diagnostics can reveal substantially higher prevalence compared to microscopy, particularly in low-intensity settings, supporting their application in the present study to improve detection of residual infections following MDA cessation [8,9,10]. Incorporating molecular diagnostics into surveillance programmes may therefore provide a more accurate assessment of residual transmission and programme effectiveness following MDA cessation.

In this context, accurate surveillance after discontinuation of MDA is essential for programme decision-making and for preventing resurgence. This study assessed the prevalence of HINIs among PSC in Anuradhapura, a sentinel site for national surveillance, using a combination of multiple microscopic techniques (wet mounts, concentration methods, KK, culture) and real-time quantitative PCR (qPCR). By integrating molecular diagnostics alongside conventional microscopy, this study also aimed to determine whether qPCR could identify additional low-intensity infections that may remain undetected by routine surveillance approaches. Conducted four years after the cessation of routine deworming, this study provides a comprehensive evaluation of residual infection status and examines the added value of molecular diagnostics in low-prevalence settings. The findings have important implications for refining surveillance strategies in countries transitioning from control to elimination of HINIs.

## 2. Materials and Methods

### 2.1. Study Area

**Figure 1:**
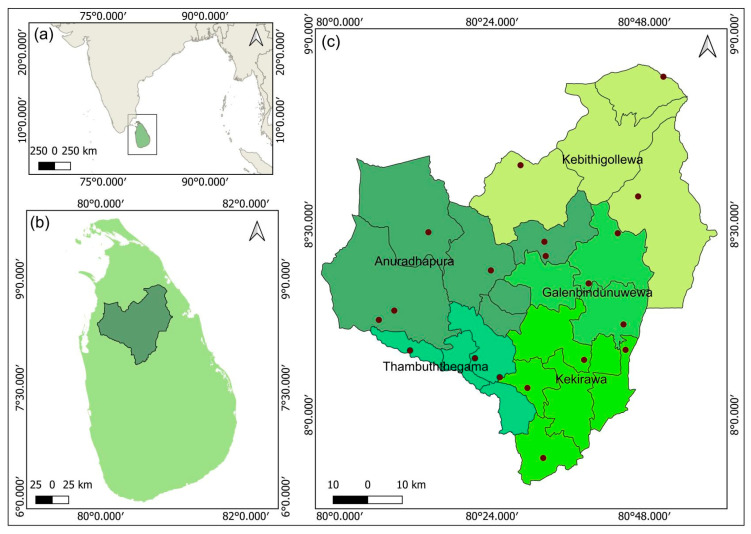
Location of the study area. (**a**) Map of South Asia indicating the position of Sri Lanka. (**b**) Map of Sri Lanka highlighting the location of the Anuradhapura District. (**c**) Map of Anuradhapura District showing the locations of the data collection sites (schools), marked by point locations. Educational zones are demarcated and colour-coded. Sub-boundaries within each zone represent the underlying administrative divisions of the district. This map served as the spatial reference for the all prevalence maps. All maps were produced using QGIS software (Version 3.40.5), with base maps obtained from the GADM database (https://gadm.org/maps/LKA/anuradhapura.html, Accessed on 20 September 2025).

Anuradhapura is one of the two districts in the North Central Province (NCP) of Sri Lanka (Figure 1), with a land extent of 7179 km^2^ (10.9% of the total area of the country). Approximately 50% of the area is covered by forests and scrublands, 22.5% by paddy fields, and 9.3% by inland water bodies [17]. The mean household income per month was approximately LKR 65,000 (217 USD) in 2019, and the poverty headcount index was 7.3% [18]. This places Anuradhapura as the district with the 12th highest household income among the 25 districts in Sri Lanka [18]. Among the total households, 46.1% obtained drinking water from protected wells, 31.4% from pipe-borne water supplies, 9.2% from rural water supply projects, and the remaining 13.3% from rivers, tanks, reservoirs, and tube wells [18]. Twenty-two per cent of the population is estimated to be urban, and 78% is rural [18]. There are five educational zones in the Anuradhapura district (Figure 1). They are Anuradhapura, Kekirawa, Thambuththegama, Galenbindunuwewa, and Kebithigollawa [19]. The district has 562 schools, including 559 state-run, with 84.2% located in rural areas. The selected study areas represent rural and semi-rural settings with mixed socioeconomic characteristics and were chosen based on their relevance to national post-MDA surveillance activities rather than strict economic classification.

### 2.2. Study Design, Setting and Population

This school-based, cross-sectional study was conducted in the Anuradhapura district of the NCP, Sri Lanka, between January and November 2023. The study population comprised PSC aged 5–11 years (Grades 1–5) enrolled in state schools. All eligible students whose parents or guardians provided informed consent were included, with no specific exclusion criteria. Deworming history was recorded using a questionnaire for descriptive purposes, but was not used as an inclusion or exclusion criterion. PSC were selected as they represent a key sentinel population for STH surveillance and are routinely targeted in national deworming programmes. This age group is widely used for monitoring transmission intensity and evaluating the impact of MDA surveillance strategies.

### 2.3. Sampling Technique and Sample Size

Sample size was calculated using the standard formula for prevalence studies (*n* = (Z_1_ − α/_2_)^2^ × *P*(1 − *P*)/d^2^), where ‘n’ is the required sample size, ‘Z’ is the critical value for a 95% confidence level of 1.96, ‘*P*’ is the expected prevalence, and ‘d’ is the margin of error [20]. Assuming a prevalence of 5% and a precision of 2% (d = 0.02), the initial sample size was 456. A design effect of 1.5 was applied to account for clustering, yielding a minimum sample size of 684. A 20% non-compliance rate for stool sample submission was initially anticipated based on previous studies [13]. However, during the early phase of field implementation, lower-than-expected stool sample return rates were observed due to field constraints such as irregular bowel habits among students and school closures, this was revised to 30%, and additional schools and students were recruited to achieve the required sample size.

A multistage stratified cluster sampling design was employed. The sampling frame consisted of all state schools with primary sections (Grades 1–5) in the Anuradhapura district, obtained from the Provincial Department of Education of the NCP [19]. In the first stage, educational zones were used as strata. The total sample allocation was distributed proportionately across zones based on the number of primary school children in each zone to ensure representativeness. In the second stage, schools within each educational zone were listed, and 19 schools were selected using simple random sampling. Random selection was performed using computer-generated random numbers in Microsoft Excel. In the third stage, within selected schools, clusters were defined based on the median class size of primary school grades. In schools with multiple classes per grade, one or more classes were selected depending on the required sample allocation.

Within each selected class, students were selected using the class register as the sampling frame. If the number of available students in a class was lower than the predefined cluster size, additional students were recruited from the next available class within the same school. In schools with only one eligible class, an additional school was randomly selected to complete the required cluster allocation. Conversely, in classes exceeding the required cluster size, students were selected by simple random sampling using the class register as the sampling frame. An equal number of students was recruited from each grade (Grades 1–5) to ensure balanced representation across primary school levels.

### 2.4. Sample Collection, Storage and Analysis

Instructions on the collection, handling, and storage of faecal samples were provided to parents or guardians, and the Scotch tape technique was demonstrated using a mannequin. Each parent/guardian received a sample collection kit containing a labelled leak-proof container, Scotch tape, disposable gloves, and written instructions outlining the collection procedure. Participants were instructed to provide one fresh faecal sample and one Scotch tape sample the following day. Upon receipt, samples were checked for labelling and adequacy of quantity and transported immediately to the Parasitology Laboratory, Faculty of Medicine and Allied Sciences, Rajarata University of Sri Lanka. In the laboratory, each faecal sample was divided into four aliquots (Supplementary File—Figure S1).

### 2.5. Faecal Microscopy

The first aliquot was processed immediately for direct saline smear (DSS), direct iodine smear (DIS), and modified KK techniques and examined within 1–2 h [21]. For KK, 41.7 mg of faeces was used per slide, which was allowed to clear for 20–60 min before being read. The mean egg count was multiplied by 24 to estimate eggs per gram (EPG) of faeces [16], and infection intensity was categorised as light, moderate, or heavy according to World Health Organisation (WHO) recommendations [16]. The second aliquot, consisting of 1 g of stool fixed in 10% formalin, was processed via the formalin ether concentration technique (FECT) to detect nematode eggs and larvae [21]. The third aliquot was analysed using agar plate culture (APC) for the detection of *Strongyloides* spp. [22], with worm identification performed according to the WHO-recommended protocol [21]. The fourth aliquot was reserved for qPCR analysis. All stool samples were processed and examined in triplicate. Scotch tape samples were examined microscopically for the detection of *E. vermicularis* eggs.

### 2.6. DNA Extraction

The stool aliquot reserved for qPCR analysis was mixed with 80% ethanol (*v*/*v*) and stored at −20 °C until completion of sample collection. DNA extraction was subsequently performed using the HiPurA Stool DNA Purification Kit (Himedia Laboratories, Mumbai, India) according to previously described procedures [23]. Briefly, 200 mg of faeces was washed with 500 µL nuclease-free water and centrifuged at 10,000 rpm for 3 min. The pellet was resuspended in 300 µL lysis solution and 20 µL proteinase K, vortexed, and incubated at 55 °C for 30 min, followed by the addition of 200 µL of stool lysis buffer and incubation at 70 °C for 10 min. To facilitate mechanical disruption of helminth egg walls, 500 mg of 0.5 mm zirconia–silicate beads was added, and the sample was homogenised using a bead homogeniser (Omni Bead Beater, Omni International, Kennesaw, GA, USA). Thereafter, 250 µL of inhibitor removal solution was added and incubated at 4 °C for 5 min, followed by centrifugation at 12,000 rpm for 1 min. The supernatant was then processed through a spin column, washed with diluted wash buffer, and the DNA was eluted in elution buffer.

To assess extraction quality, 10% of the DNA samples were analysed using a microvolume UV–Vis spectrophotometer (NanoDrop 2000, Thermo Scientific, Waltham, MA, USA). Extracted DNA was stored at −20 °C until shipment to the QIMR Berghofer Medical Research Institute, Australia, where qPCR analysis was performed. Samples were transported on dry ice and stored at −20 °C upon arrival until analysis.

### 2.7. Multiplex qPCR

Three qPCR assays were performed: a triplex assay targeting *A. lumbricoides*, *T. trichiura*, and *N. americanus*; a duplex assay for *A. duodenale* and *A. ceylanicum*; and singleplex assays for *Strongyloides* spp. and *S. stercoralis* separately. The assays utilised previously published primers and probes (Table 1). All reactions were performed in a 15 μL total volume containing 8 μL GoTaq master mix (Promega Corporation, Madison, WI, USA), 2 μL template DNA, nuclease-free water, and the appropriate concentrations of primers and probes (Table 1). Using the CFX384 real-time PCR system (Bio-Rad, Hercules, CA, USA), qPCR was performed under the following cycling conditions: 95 °C for 3 min, followed by 40 cycles of 95 °C for 10 s, 55 °C for 30 s, and 72 °C for 30 s, with a final extension at 72 °C for 5 min. All samples were run in triplicate. Each run included standard positive controls and no-template controls (NTCs). Positive controls consisted of synthetic gene fragments (gBlocks; IDT) for each target species, prepared as a 1:10 serial dilution series. Samples were considered positive if amplification occurred in at least two of three replicates, with an average cycle threshold (Ct) < 35 and Ct standard deviation ≤ 1. Microscopy results were blinded to the qPCR analysis.

### 2.8. Sanger Sequencing and Sequence Analysis

All *Strongyloides* spp. qPCR-positive samples targeting the small subunit ribosomal RNA (SSU rRNA) were sequenced to confirm species identity. Sequencing reactions included 6 pmol of primer and 5 ng of PCR product per 100 bp of expected amplicon, adjusted to a final volume of 9.3 µL with nuclease-free water. Reactions were processed at the QIMR Berghofer sequencing facility (Brisbane, QLD, Australia), beginning with a 5 min incubation at 96 °C, followed by the addition of 0.6 µL BigDye terminator and 2.1 µL 5× sequencing buffer, and subsequent thermocycling. Amplicons were purified by centrifugation with 70% isopropanol at 4 °C, washed, and dried.

Raw sequences were analysed using Geneious Prime (v2024.0.7; Biomatters Ltd., Auckland, New Zealand). Sequences were trimmed, contigs assembled de novo, and discordant bases manually corrected. Consensus sequences were extracted and compared against the NCBI GenBank nucleotide database (nr/nt) using BLAST (version 2.17.0) to determine the species identity.

### 2.9. Data Quality Assurance and Analysis

Faecal samples were transported to the Medical Parasitology Laboratory, Rajarata University of Sri Lanka, within two hours without preservative. To minimise observer bias, triplicate slides were examined independently by three experienced microscopists, and the results were recorded separately. Discordant findings were reviewed by the principal investigator. Ten per cent of samples were randomly re-examined by experienced laboratory technologists who did not have prior knowledge of the results. Microscopists were blinded to the results of all other methods. The data were entered into Microsoft 365 Excel and analysed via the Statistical Package for Social Sciences (SPSS) version 25.

Individuals were considered positive for HINIs if at least one replicate from any microscopic method (DSS, DIS, KK, FECT, APC, or Scotch tape) detected eggs, larvae, or adult worms or if qPCR Ct average was <35 with SD ≤ 1. Prevalence was calculated using descriptive statistics (frequency, percentages with 95% CIs, means and SD), and the associations were tested via the chi-square test. Assumptions for chi-square analysis were evaluated based on expected cell counts, and Fisher’s exact test was applied when more than 20% of cells had expected counts <5 or when chi-square assumptions for asymptotic validity were not satisfied. Infection intensity was expressed as the arithmetic mean EPG of faeces.

Sensitivity and specificity were calculated using species-specific reference standards (KK for *A. lumbricoides*, *T. trichiura* and hookworms, APC for *Strongyloides* spp. and Scotch tape test for *E. vermicularis*) and a composite reference standard derived from all diagnostic tests. These metrics were calculated from 2 × 2 contingency tables of true positives, true negatives, false positives, and false negatives, with 95% CI reported.

Prevalence estimates used method-specific denominators to maximise accuracy and representativeness. Microscopy-based HINI prevalence included all individuals who underwent all the microscopic tests, including the Scotch tape test for *E. vermicularis*. Prevalence estimates for the qPCR combined method comparison were restricted to individuals with adequate samples tested by both qPCR and all microscopic methods, ensuring consistent and inclusive denominators for reliable estimation and evaluation of fair test performance.

## 3. Results

Between January and November 2023, 967 PSC were recruited from 19 schools. A total of 781 students provided Scotch tape samples, and of them, 688 provided faecal samples as well (Supplementary file—Table S1). Microscopic examination was carried out on all 688 samples, while qPCR was carried out on 626 samples, depending on the adequacy of the remaining sample volume. The mean age of the study participants was 7.9 years (SD ± 1.44), and 56.5% were boys.

### 3.1. HINI Prevalence and Intensity

Prevalence estimates were calculated using method-specific denominators, with microscopy-based estimates derived from all stool samples (*n* = 688), *E. vermicularis* microscopy prevalence based on Scotch tape samples (*n* = 781), and qPCR-based and combined estimates with samples having results available from both qPCR and microscopy results (*n* = 626).

#### 3.1.1. Microscopic Prevalence

Among 688 samples examined by microscopy, overall STH prevalence was 11.6% (95% CI: (9.4–14.2); *n* = 80). Parasite-specific prevalences were highest for *A. lumbricoides* (5.7%; 95% CI: 4.2–7.7; *n* = 39) and *Strongyloides* spp. (5.2%; 95% CI: 3.8–7.1; *n* = 36). *E. vermicularis* demonstrated the highest microscopic prevalence among all HINIs, at 24.5% (95% CI: 21.4–27.8; *n* = 191). Overall, the microscopic prevalence of HINIs was 32.4% (95% CI: 29–36; *n* = 223), calculated based on the total sample of 688 individuals who were examined using all microscopy-based methods, including APC and the Scotch tape test (Table 2).

#### 3.1.2. Molecular Prevalence

Among 626 samples analysed by qPCR, STH prevalence was 37.2% (95% CI: 33.5–41.1; *n* = 233). Species-specific prevalences were highest for *S. stercoralis* (16.1%; 95% CI: 13.4–19.2; *n* = 101), *A. lumbricoides* (15.7%; 95% CI: 13.1–18.8; *n* = 98). Overall molecular prevalence for HINIs was not specifically estimated, as *E. vermicularis* was not assessed by qPCR (Table 2). Sequencing of *Strongyloides* spp. positive samples confirmed that all infections were caused by *S. stercoralis*, with all analysed sequences showing 100% identity to reference sequences of *S. stercoralis*.

#### 3.1.3. Combined Prevalence

In 626 samples analysed by all microscopic tests and qPCR, the combined overall STH prevalence was 41.7% (95% CI: 37.9–45.6; *n* = 261). Species-specific prevalences were highest for *S. stercoralis* (19.2%; 95% CI: 16.3–22; *n* = 120), and *A. lumbricoides* (18.1%; 95% CI: 15.2–21.3; *n* = 113), followed by hookworms (6.9%; 95% CI: 5.1–9.1; *n* = 43), and *T. trichiura* (6.4%; 95% CI: 4.7–8.1; *n* = 40). With inclusion of *E. vermicularis* detected by microscopy, the combined overall prevalence of HINIs in this subset was 54.2% (95% CI: 50.2–58; *n* = 339) (Table 2).

**Figure 2:**
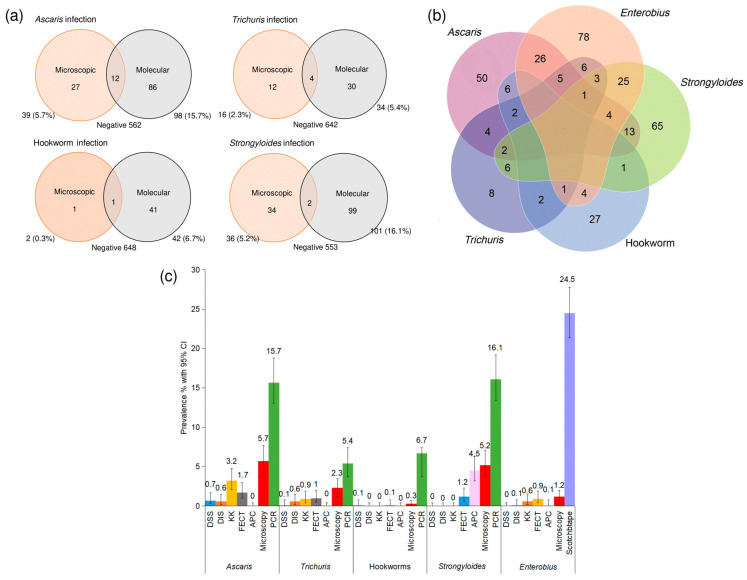
Prevalence and coinfection of human intestinal nematode infections (HINIs) among the participants (*n* = 626). (**a**) Prevalence of soil-transmitted helminths by microscopy and qPCR. (**b**) Coinfection among the participants. (**c**) Prevalence of HINIs with different diagnostic methods. *E. vermicularis* was not tested with molecular analysis. Only *E. vermicularis* was assessed via Scotch tape analysis.

All infections detected by the KK method were of low intensity. STH prevalence did not differ between males and females (χ^2^ = 3.30, df = 1, *p* = 0.069), with prevalences of 44.8% (95% CI: 36.1–48.3) and 37.6% (95% CI: 32.0–41.1), respectively. *E. vermicularis* prevalence was 22% (95% CI: 18.03–26.64%) among females, and 27.6% (95% CI: 22.60–33.17%) among males, with no evidence of association by gender and infection (χ^2^ = 2.26, df = 1, *p* = 0.132) (Figure 2a).

### 3.2. Coinfections

Polyparasitism assessed using combined microscopy and qPCR (*n* = 626) was observed in 32.7% (95% CI: 28–37.9, *n* = 111) of infected individuals. Dual infections predominated (83.8%; 95% CI: 75.8–89.5, *n* = 93), followed by triple infections (15.3%; 95% CI: 9.8–23.2, *n* = 17). A single case of quadruple infection (0.9%; 95% CI: 0.2–4.9, *n* = 1) was identified, involving *A. lumbricoides*, *T. trichiura*, *S. stercoralis*, and *E. vermicularis*. The most frequent coinfection combination was *A. lumbricoides* and *E. vermicularis* (32.4%, 95% CI: 24.4–41.6; *n* = 36), followed by *S. stercoralis* and *E. vermicularis* (29.7%; 95% CI: 22–38.8; *n* = 33) (Figure 2b).

### 3.3. Geographic Variation in STH and E. vermicularis Prevalence

**Figure 3:**
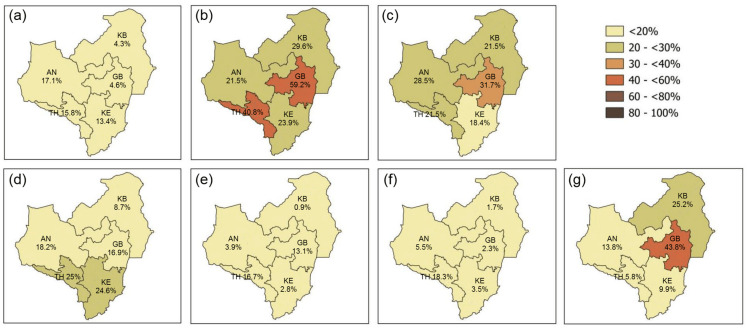
Map of Anuradhapura District showing (**a**) microscopic prevalence of soil-transmitted helminth infections (*n* = 688), (**b**) qPCR prevalence of soil-transmitted helminth infections (*n* = 626), (**c**) *E. vermicularis* infection prevalence (Scotch tape method) (*n* = 781), (**d**) combined *A. lumbricoides* prevalence, (**e**) combined *T. trichiura* prevalence, (**f**) combined hookworm prevalence, and (**g**) combined *S. stercoralis* prevalence according to the educational zone. Demarcations within the district indicate the educational zone boundaries. The values indicate the prevalence of each educational zone. For combined prevalence, *n* = 626. Educational zones: AN, Anuradhapura; TH, Thambuththegama; KE, Kekirawa; GB, Galenbindunuwewa; KB, Kebithigollewa.

STH and *E. vermicularis* prevalences were stratified by educational zone (Figure 3 and Supplementary file—Table S2). STH prevalence was highest in the Galenbindunuwewa educational zone (62.3%, 95% CI 54–70.6) and lowest in the Anuradhapura educational zone (31.5%, 95% CI 24.7–38.3). There was a significant association between geographic location and infection status (χ^2^ = 40.5, df = 4, *p* < 0.0001) with a moderate effect size (Cramér’s V = 0.24). *E. vermicularis* prevalence was also highest in Galenbindunuwewa educational zone (31.7%, 95% CI 46.1–67.9) and lowest in Kekirawa educational zone (18.4%, 95% CI 12.6–24.2). There was no statistically significant association between the geographical location and the prevalence of *E. vermicularis* infection (χ^2^ = 9.07, df = 4, *p* = 0.059).

At the school level, STH prevalence ranged from 4.3% to 73.3%, with the highest prevalence observed in a school from the Galenbindunuwewa educational zone and the lowest in a school from the Kekirawa educational zone (Supplementary file—Table S2). For *E. vermicularis*, school-level prevalence ranged from 7.5% to 41.0%, with the highest prevalence recorded in a school from the Galenbindunuwewa educational zone and the lowest in a school from the Thambuttegama educational zone.

### 3.4. Parasite-Specific Comparison of Microscopic Methods

A comparison of the microscopic methods (*n* = 688) revealed distinct detection rates for each STH. For *A. lumbricoides*, KK identified the highest number of positive samples (*n* = 22; 3.2%; 95% CI: 1.9–4.5), followed by the FECT (*n* = 12; 1.3%; 95% CI: 0.5–2.2). In contrast, FECT was the most sensitive method for detecting *T. trichiura*, yielding nine positive samples (1.3%, 95% CI: 0.3–1.8), compared to six (0.9%, 95% CI: 0.2–1.6) by KK. Hookworm infections were the least frequently detected by microscopy. The two cases were identified by FECT and DWS. Both the KK method and DIS did not detect any hookworm infections. For *Strongyloides* spp., APC detected the highest number of positives (*n* = 31; 4.5%; 95% CI: 3.2–6.3) followed by FECT (*n* = 5; 0.7%; 95% CI: 0.3–1.7). All other microscopic methods failed to detect any positive instances of the parasite (Figure 2c).

### 3.5. Species-Specific Comparison of Microscopy and qPCR

Among the 626 samples analysed by both methods, qPCR demonstrated higher detection rates across all parasites, identifying 98 (15.7%) *A. lumbricoides* infections compared with 27 (4.3%) by microscopy, 34 (5.4%) vs. 10 (1.6%) for *T. trichiura*, 42 (6.7%) vs. 2 (0.3%) for hookworm, and 101 (16.1%) vs. 21 (3.4%) for *S. stercoralis* (Figure 2a).

### 3.6. Comparative Diagnostic Performance of All Microscopic Methods and qPCR

**Figure 4:**
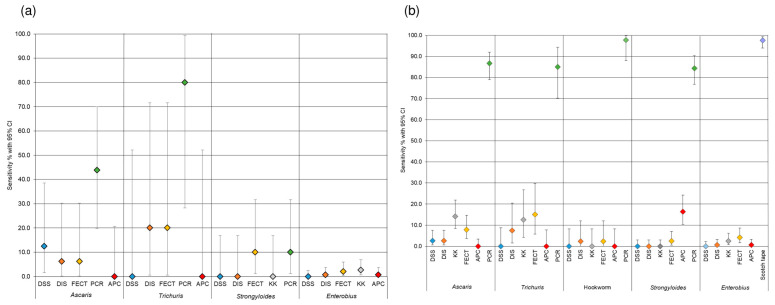
Sensitivity of different diagnostic methods for detecting human intestinal nematode infections. For soil-transmitted helminths, a total of 626 samples were analysed using both microscopy and qPCR. For *E. vermicularis* detection, 688 samples were examined using all microscopic methods, including the Scotch tape technique. (**a**) Species-specific reference standard: KK for the *A. lumbricoides*, *T. trichiura* and hookworm infections, APC for *Strongyloides* spp. infection, and the Scotch tape test for *E. vermicularis* infection. Sensitivity values for hookworm infection were not assessed, as no individuals were positive with KK; (**b**) Composite reference standard: comprised all the microscopic methods and qPCR for STH infections. All the microscopic methods, including the Scotch tape test, were used as the reference for *E. vermicualris* infection. Whiskers indicate the 95% confidence interval.

Diagnostic accuracy was evaluated in the subset of 626 samples tested by all microscopic methods and qPCR. When evaluated against species-specific reference standards (KK for *A. lumbricoides*, *T. trichiura* and hookworms, APC for *S. stercoralis* and Scotch tape test for *E. vermicularis*), the sensitivity of diagnostic methods varied considerably across helminth species (Supplementary file—Tables S3–S8). For *A. lumbricoides*, qPCR demonstrated the highest sensitivity (43.8%), while conventional stool-based methods showed lower sensitivities, including DSS (12.5%) and DIS and FECT (6.3% each). Similarly, for *T. trichiura*, PCR showed superior sensitivity (80.0%), whereas DIS and FECT each demonstrated moderate sensitivity (20.0%), and DSS failed to detect any infections. For *Strongyloides* spp. PCR and FECT showed comparable sensitivities (10.0%) relative to agar plate culture, while DSS, DIS, and KK did not detect any positive cases. In the case of *E. vermicularis,* all the methods showed markedly lower sensitivities, including KK (2.7%), FECT (2.0%), and DIS and APC (0.7% each). Sensitivity for hookworm could not be determined, as no positive cases were identified by the KK method (Figure 4).

When evaluated against a composite reference standard (any positive result by any method), the sensitivity estimates increased substantially across all parasites. Across *A. lumbricoides*, *T. trichiura*, and hookworms, qPCR demonstrated the highest sensitivity, with the values of 86.7%, 85%, and 97.7%, respectively. A similar pattern was observed for *S. stercoralis*, where qPCR (84.4%) outperformed traditional microscopy. Of the microscopic methods, KK was the most sensitive for *A. lumbricoides* (14.2%), FECT for *T. trichiura* (15.0%), DIS and FECT for Hookworm (2.3%), and APC for *Strongyloides* spp. (16.4%), and the Scotch Tape method for *E. vermicularis* (97.6%) (Figure 4).

Across all species, all microscopic methods consistently demonstrated high specificity (98.2–100%). PCR, in contrast, showed 100% specificity for hookworm but slightly lower specificities for *A. lumbricoides* (85.1%), *S. stercoralis* (83.7%), and *T. trichiura* (95.2%) when assessed against the microscopic reference standard. When compared against a composite reference standard, all diagnostic methods, including PCR, achieved 100% specificity.

## 4. Discussion

This study provides the first molecular assessment of STH infections in a setting previously classified as low-endemic, based on routine microscopy [13]. Reliance on conventional diagnostics alone can substantially underestimate infection prevalence, particularly in low-intensity transmission settings, with important implications for decision-making in programme implementation [28]. This school-based study, conducted among PSC using a multistage stratified cluster design, provides updated epidemiological insights for STH and *E. vermicularis* infections. The overall combined prevalence of STH infections was 41.7%, while the prevalence of HINIs (STH and *E. vermicularis*) was 54.1%. Polyparasitism was observed in 32.7% of infected individuals. All infections detected by the KK method were of light intensity, indicating ongoing transmission at low infection intensities. Although adults were not included in this study, the persistence of infections among schoolchildren, who are the primary targets of MDA, suggests continued transmission within the wider population. This is important in terms of preventive strategies, as school-based surveys form the basis of national deworming policy decisions [15].

The performance gap between diagnostic methods was substantial. The KK method, the WHO-recommended standard for STH surveys, detected only 16 of 113 *A. lumbricoides*, 5 of 40 *T. trichiura*, and none of 42 hookworm infections identified by qPCR, demonstrating marked underdetection. Even when multiple microscopic methods were combined, a substantial proportion of infections remains undetected. Compared to microscopy, qPCR detected approximately 3.6-fold more *A. lumbricoides*, 3.4-fold more *T. trichiura*, 21-fold more hookworm, and 4.8-fold more *S. stercoralis* infections, reflecting the intrinsic differences between the diagnostic approaches. These findings are consistent with reports from other low-endemic settings, where microscopy performs poorly for low-intensity infections [8,9,10]. Microscopy depends on the visual detection of intact eggs or larvae, and is influenced by infection intensity, timing, stool volume, and sample integrity, whereas qPCR detects parasite DNA, even when egg output is low, intermittent, or degraded [6,29]. This limitation was particularly evident for *S. stercoralis* and hookworm, where fragile eggs, larvae, and low parasite burdens reduce microscopy sensitivity [28]. Consequently, KK-based prevalence thresholds may be insufficiently sensitive to guide preventive strategies in near-elimination settings [28].

However, these findings do not support universal replacement of microscopy with molecular diagnostics. While qPCR offers superior sensitivity, its cost, infrastructure requirements, and technical complexity limit routine implementation in many low and middle-income settings, particularly when considered at national-scale surveillance, where recurrent costs for reagents, equipment maintenance, trained personnel, and quality assurance become substantial. Conversely, microscopy remains operationally feasible but diagnostically limited in low-intensity contexts [30]. A tiered, context-specific surveillance strategy can therefore be recommended, combining enhanced microscopy (e.g., multiple smears, FECT, Mini-FLOTAC) for routine use with targeted molecular assessments at sentinel sites or key decision points, such as prior to modification of MDA programmes [28]. School-based platforms provide a practical and feasible structure for such integrated surveillance.

Our findings also highlight important gaps in current surveillance strategies. *E. vermicularis* was the most prevalent infection (24.5%), exceeding global estimates of 12% and falling at the upper end of reported regional ranges (5–20%) [31,32]. *E. vermicularis* is currently not included in the national and global deworming surveillance targets, which prioritise *A. lumbricoides*, *T. trichiura*, and hookworms. Reliance on stool-based diagnostic methods would have resulted in substantial underdetection of *E. vermicularis* infections, as demonstrated by the cases identified using the Scotch tape method in this study. This is primarily attributable to the parasite’s biological pattern of perianal egg deposition, which limits the presence of eggs in faecal samples compared with other intestinal helminths. As a result, routine faecal smear-based surveillance is likely to underestimate the true prevalence of infection. However, the Scotch tape test offers a highly sensitive (90% with three consecutive sampling) and cost-effective (USD 0.40–1.00 consumable cost per person) alternative [31,32]. Integrating this simple diagnostic into existing surveillance systems would facilitate comprehensive parasite monitoring with minimal additional resource requirements.

Similarly, *S. stercoralis* was detected at a prevalence of 16.1% by qPCR (5.2% by microscopy), substantially higher than prior estimates. Previous community-based studies in Sri Lanka reported much lower prevalence rates of 0.5% and 2.1% in the Northern Province (1986, 1989) and 0.9% in Sabaragamuwa Province (2006) [33,34,35]. A more recent study (2024) among immunocompromised populations recorded prevalences of 16.4% using a combination of microscopy, serology, and PCR. This likely reflects both improved detection and longstanding under-recognition [36]. Importantly, standard MDA regimens (albendazole, mebendazole) have limited efficacy against *Strongyloides* spp. [37,38], and failure to detect this infection may undermine control efforts. According to the 2024 WHO guideline, countries should conduct prevalence surveys on *Strongyloides* spp. using sensitive coprological methods such as the Baermann technique or APC, integrating them into national NTD control whenever possible [37,38]. In endemic settings where the prevalence among school-aged children is ≥5%, WHO conditionally recommends annual MDA with single-dose ivermectin (200 µg/kg) targeting the entire community aged five years and above for at least five years, followed by reassessment [37,38]. The microscopic prevalence observed in the present study exceeds this threshold, suggesting that *S. stercoralis* infection may represent an under-recognised public health problem in this area. Sequencing of *Strongyloides* spp. positive products confirmed that the infections were caused by *S. stercoralis*, ruling out any potential zoonotic origin such as *S. fuelleborni* which has been identified to cause human infections in Asia [39]. This finding is particularly important in Anuradhapura, where non-human primates are abundant and frequently share farmland with humans, unlike in other regions of the country [40]. Collectively, these findings suggest that post-MDA surveillance and deworming strategies may need to expand beyond conventional STH targets to incorporate species-specific monitoring and control approaches for neglected infections such as *E. vermicularis* and *S. stercoralis*.

Importantly, the overall microscopic prevalence of *A. lumbricoides*, *T. trichiur* and hookworm infections among PSC in this study (4.2% by triplicate KK) was notably higher than the 0.21% reported in the 2017 national survey, where only hookworms were detected in Anuradhapura. This observed difference should be interpreted with caution due to key methodological differences between the two surveys, including variation in sampling design (school grade-specific PSC cohorts), sample size, and diagnostic protocols (duplicate versus triplicate KK). These factors are known to influence detection sensitivity, particularly for low-intensity infections. Therefore, while the findings may suggest a higher detected prevalence in the present study, direct inference of temporal trends or epidemiological change cannot be made with confidence. Notwithstanding these limitations, the use of triplicate KK in the present study likely improved diagnostic sensitivity compared with the 2017 survey, particularly for light-intensity infections. In addition, potential epidemiological changes following the scale-back of MDA in low-risk districts such as Anuradhapura may also have contributed to the observed pattern, although this cannot be confirmed based on available comparative data.

Molecular analysis further enabled species-level identification, confirming the presence of *A. ceylanicum* in the population for the first time in a prevalence survey, while *N. americanus* remained the dominant hookworm species [41]. Notably, no *A. duodenale* infections were detected, consistent with previous reports [14]. By using qPCR, this study not only demonstrates the superior sensitivity of molecular diagnostics over traditional microscopy but also provides critical species-level information that was not available from community-based surveys in Sri Lanka. These findings have important insights into transmission dynamics, zoonotic risks, and implications for targeted control strategies.

Several limitations should be considered. Although sample transport from schools to the laboratory was implemented to minimise processing delays, stool collection timing could not be standardised, as some children passed stool at different times of the day. This unavoidable variability may have resulted in differential delays before processing, during which fragile parasite stages, particularly hookworm eggs, may have been affected, potentially reducing microscopy sensitivity. Use of a single stool sample may have resulted in an underestimated prevalence due to intermittent egg shedding, particularly for microscopy [42]. Similarly, a single adhesive tape test (~50% sensitivity) likely underestimated *E. vermicularis* detection compared with repeated sampling (~90% sensitivity) [31,32]. The reduced number of samples subjected to qPCR due to limited remaining stool material represents a methodological limitation; however, since exclusion was determined by sample availability rather than infection status or participant characteristics, any potential selection bias is likely to be minimal. Although qPCR demonstrated higher sensitivity overall, some microscopically positive samples were qPCR-negative, possibly due to low parasite DNA concentrations, extraction inefficiencies, or PCR inhibitors. While bead-beating was used to improve egg disruption and DNA extraction, limitations persist for *T. trichiura* [10,43]. Dilution (1:5) of extracted DNA reduced inhibitors, but may have lowered concentration in low-intensity infections, increasing Ct values, and risk of false negatives [44].

Taken together, these findings highlight key epidemiological and surveillance implications for HINIs in a post-deworming setting. While the greater sensitivity of molecular diagnostics compared with microscopy is well established, the present study provided novel molecular epidemiological data in this setting. Importantly, this study was conducted in a post-MDA, low-endemic context where long-term deworming programmes had led to the assumption that transmission had declined to very low levels and scaling down of the regular school-based deworming. By applying qPCR alongside multiple microscopic techniques, this study demonstrates that a substantial burden of sub-patent infections persists despite previously reported very low prevalence. The higher prevalence observed suggests that transmission persists, possibly reflecting the cessation of routine deworming programmes in districts classified as low risk. These findings support periodic prevalence reassessment as control programmes transition from control to elimination. Microscopy alone likely underestimates the true burden due to low-intensity and intermittent infections. Integrating more sensitive diagnostic approaches, such as PCR, can improve detection of residual transmission and guide programme strategies. This study provides important baseline molecular data, identifies under-recognised infections such as *S. stercoralis*, and confirms *Ancylostoma* spp. More broadly, as countries approach elimination and infection intensities decline, surveillance must evolve to incorporate more sensitive diagnostics to accurately capture transmission and avoid premature relaxation of control efforts.

Deworming programmes should not be scaled back solely on microscopy-derived low prevalence; periodic reassessment using integrated diagnostics is essential to avoid persistent or resurgent transmission. In alignment with our findings and the WHO 2030 roadmap for NTDs, we propose context-specific recommendations to strengthen surveillance and guide elimination-oriented strategies for intestinal nematode infections in post-MDA settings, including integration of molecular diagnostics, expanded parasite coverage, and targeted sentinel approaches (Table 3). Globally, as countries transition towards elimination, integrated surveillance combining improved microscopy and targeted molecular diagnostics is important for accurately defining residual transmission and guiding control strategies. Within this broader context of WASH-supported control programmes, these findings are particularly relevant for strengthening surveillance frameworks in endemic settings. However, WASH-related environmental and behavioural determinants were not assessed in the present analysis; therefore, their contribution to infection patterns could not be evaluated. Operationally, such enhanced surveillance could be feasibly implemented through existing school health platforms and periodic national surveys, with phased or cyclical reassessment (e.g., aligned with routine national survey cycles every few years), enabling sustainable monitoring of post-MDA transmission dynamics. Importantly, beyond confirming higher sensitivity, this study demonstrates how diagnostic choice directly influences public health decision-making within a real-world, school-based surveillance framework.

## 5. Conclusions

This is the first molecular prevalence assessment of STH infections in Sri Lanka, revealing a markedly higher prevalence than previously reported in a setting considered low-endemic. A substantial underestimation of HINIs, including STH infections, can occur when surveillance relies solely on conventional microscopy in low-endemic settings. The higher prevalence detected using combined diagnostic approaches suggests ongoing low-intensity transmission, potentially compounded by strategic changes such as the scale-back of mass deworming. The key implication is not the universal replacement of microscopy with molecular tools, but the need for integrated, context-specific surveillance strategies that combine feasibility with diagnostic sensitivity. Periodic incorporation of molecular methods into surveillance frameworks can improve accuracy at critical decision points, particularly in settings approaching elimination. Such enhanced surveillance could be operationally integrated into existing school health systems and periodic national surveys, enabling phased implementation aligned with routine surveillance cycles. The presence of low-intensity infections and the high prevalence of species like *S. stercoralis* and *E. vermicularis* highlight the gaps in current control programmes and the need for more inclusive surveillance and diagnostic strategies. Globally, these findings emphasise that as countries transition towards low endemicity, surveillance systems must adapt to avoid premature policy decisions based on insensitive diagnostics. Continuous laying out in targeted interventions, supported by improved diagnostics, remains essential for accurately identifying residual transmission and guiding control strategies for STH infections within integrated control programmes.

## Figures and Tables

**Table 1:** Primers and probes for the qPCR assays.

Assay Type	Species	Target		Forward primer	Final Concentration (nM)	Fluorophore	Reference
Triplex	*Necator americanus*	ITS2	F	CCAGAATCGCCACAAATTGTAT	200	HEX	[24]
			R	GGGTTTGAGGCTTATCATAAAGAA	200		
			P	CCCGATTTGAGCTGAATTGTCA AA	100		
	*Trichuris trichiura*	ITS1	F	GGCGTAGAGGAGCGATTT	60	ROX	[25]
			R	TACTACCCATCACACATTAGCC	60		
			P	TTTGCGGGCGAGAACGGAAATATT	100		
	*Ascaris lumbricoides*	ITS1	F	GTAATAGCAGTCGGCGGTTTCTT	60	FAM	[25]
			R	GCCCAACATGCCACCTATTC	60		
			P	TTGGCGGACAATTGCATGCGAT	100		
Duplex	*Ancylostoma c* *e* *ylanicum*	Repeat	F	CAAATATTACTGTGCGCATTTAGC	200	FAM	[26]
			R	GCGAATATTTAGTGGGTTTACTGG	200		
			P	CGGTGAAAGCTTTGCGTTATTGCGA	200		
	*Ancylostoma duodenale*	Repeat	F	GTATTTCACTCATATGATCGAGTGTTC	200	Cy5	[25]
			R	GTTTGAATTTGAGGTATTTCGACCA	200		
			P	TGACAGTG TGTCATACTGTGGA AA	200		
Singleplex	*Strongyloides stercoralis*	Repeat	F	GGGCCGGACACTATAAGGAT	100	FAM	[25]
			R	TGCCTCTGGATATTGCTCAGTTC	100		
			P	ACACACCGGCCGTCGCTGC	100		
Singleplex	*Strongyloides* spp.	SSU rRNA	F	GGGCCGGACATATAAGGAT	100	Cy5	[27]
			R	TGCCTCTGGATATTGCTCAGTTC	100		
			P	ACACACCGGCCGTCGCTGC	100		

ITS1; internal transcribed spacer 1, ITS2; internal transcribed spacer 2, SSU rRNA; small subunit ribosomal ribonucleic acid, F; forward primer, R; reverse primer, P; probe, Ref; reference.

**Table 2:** Prevalence of human intestinal nematode infections detected by microscopy and qPCR.

Parasitic Species	Prevalence (%) with 95% Confidence Interval, and the Number Detected		
	Microscopy (Total Tested = 688)	qPCR (Total Tested = 626)	Combined Prevalence (Total Tested = 626)
*A. lumbricoides*	5.7 (4.2–7.7), 39	15.7 (13.1–18.8), 98	18.1 (15.2–21.3), 113
*T. trichiura*	2.3 (1.4–3.7), 16	5.4 (3.9–7.5), 34	6.4 (4.7–8.6), 40
*N. americanus*	0.3 (0.1–1.1), 2	5.4 (3.9–7.5), 34	6.9 (5.1–9.1), 43
*A. duodenale*		0 (0.0–0.5), 0	
*A. ceylanicum*		1.3 (0.7–2.5), 8	
*Strongyloides* spp *	5.2 (3.8–7.1), 36	16.1 (13.4–19.2), 101	19.2 (16.3–22), 120
*E. vermicularis* ^#^	24.5 (21.4–27.8), 191	–	–
Overall STH	11.6 (9.4–14.2), 80	37.2 (33.5–41.1), 233	41.7 (37.9–45.6), 261
Overall HINIs	32.4 (29–36), 223	–	54.2 (50.2–58), 339

* For *Strongyloides* spp. qPCR confirmed *S. stercoralis*; ^#^ Number of Scotch tape samples examined by microscopy was 781.

**Table 3:** Strategic recommendations for surveillance and control of intestinal nematode infections in post-MDA settings.

Domain	Priority Actions	Rationale and Alignment to the WHO 2030 Roadmap
Reassessment of national STH burden	Conduct a national, school-based cross-sectional survey	Substantial data gaps (For Sri Lanka, ~8 years since the last national survey and ~6 years since scale-back of preventive chemotherapy), supporting the need for adaptive monitoring to guide control strategies in transition settings [45,46]
Expanded parasite surveillance (*Enterobius*)	Integrate *E. vermicularis* into routine surveillance (adhesive tape method)	Likely under-recognised despite evidence of persistence, *E. vermicularis* should be integrated into school-based surveillance (e.g., adhesive tape testing) to better define the overall HINI burden beyond conventional STH targets [45,46]
Targeted integration of molecular diagnostics	Deploy PCR in sentinel sites, particularly in low-prevalence and post-MDA areas (complementary to microscopy)	Targeted molecular approaches are critical for detecting low-intensity infections sustaining residual transmission following cessation of preventive chemotherapy; sentinel-site implementation offers a cost-effective strategy aligned with recommendations for sensitive diagnostics in elimination settings [7,28,37,45]
Strengthening *Strongyloides* surveillance	Initiate targeted mapping using Baermann, culture ± PCR	A major evidence gap exists for strongyloidiasis; targeted surveillance is needed to generate baseline data, inform potential inclusion in control programmes, and guide policy decisions on access to ivermectin [38,45,47,48]
Integration of KAP assessments	Conduct parallel KAP surveys among caregivers and schoolchildren	Identification of gaps in knowledge, hygiene practices, and behavioural risk factors is essential to guide targeted interventions, consistent with WHO 2030 priorities on community engagement and behaviour change [45]
WASH assessment and integration	Evaluate and strengthen WASH infrastructure in schools and households in sentinel areas.	Persistent transmission is linked to environmental exposure; strengthening WASH infrastructure based on local assessments is essential, in line with WHO recommendations for integrating WASH with chemotherapy to interrupt transmission [45,49]

## Data Availability

The original contributions presented in this study are included in the article and supplementary materials. Further inquiries can be directed to the corresponding authors.

## Supplementary Materials

The following supporting information can be downloaded at: https://www.mdpi.com/article/10.3390/tropicalmed11060147/s1. **A1. Supplementary methods** Summary of faecal sample examination process, Figure S1 The examination process of the faecal samples. **A2. Supplementary results** Table S1. Details of the number of students recruited and samples received Table S2. Prevalence of soil-transmitted helminths and *E. vermicularis* according to the educational zone Table S3. Positivity by different examination methods Table S4. Sensitivity and specificity of the diagnostic methods in detecting ascariasis Table S5. Sensitivity and specificity of the diagnostic methods in detecting trichuriasis Table S6. Sensitivity and specificity of the diagnostic methods in detecting hookworm infection Table S7. Sensitivity and specificity of the diagnostic methods in detecting strongyloidiasis Table S8. Sensitivity and specificity of the diagnostic methods in detecting enterobiasis.
