# Supplementary material for: Residual Human Intestinal Nematode Infections Following Discontinuation of Mass Drug Administration in a Rural South Asian Setting: Implications for Deworming Surveillance
Source: Trop Med Infect Dis. 2026 May 27;11(6):147. doi: 10.3390/tropicalmed11060147 (PMC13307587; doi:10.3390/tropicalmed11060147)
Supplement: Supplementary file 1 [file tropicalmed-11-00147-s001.zip › tropicalmed-4280527-supplementary.pdf]

Content **Page no**

A1. Supplementary methods

1. Summary of faecal sample examination process 2

A2. Supplementary results

1. Table 1. Details of the number of students recruited and samples received 2
2 Table 2. Prevalence of soil-transmitted helminths and *E. vermicularis* according to the educational zone 3
3. Table 3. Positivity by different examination methods 4
4. Table 4. Sensitivity and specificity of the diagnostic methods in detecting ascariasis 4
5. Table 5. Sensitivity and specificity of the diagnostic methods in detecting trichuriasis 5
6. Table 6. Sensitivity and specificity of the diagnostic methods in detecting hookworm infection 5
7. Table 7. Sensitivity and specificity of the diagnostic methods in detecting strongyloidiasis 6
8. Table 8. Sensitivity and specificity of the diagnostic methods in detecting enterobiasis 6

A 1. Supplementary Methods

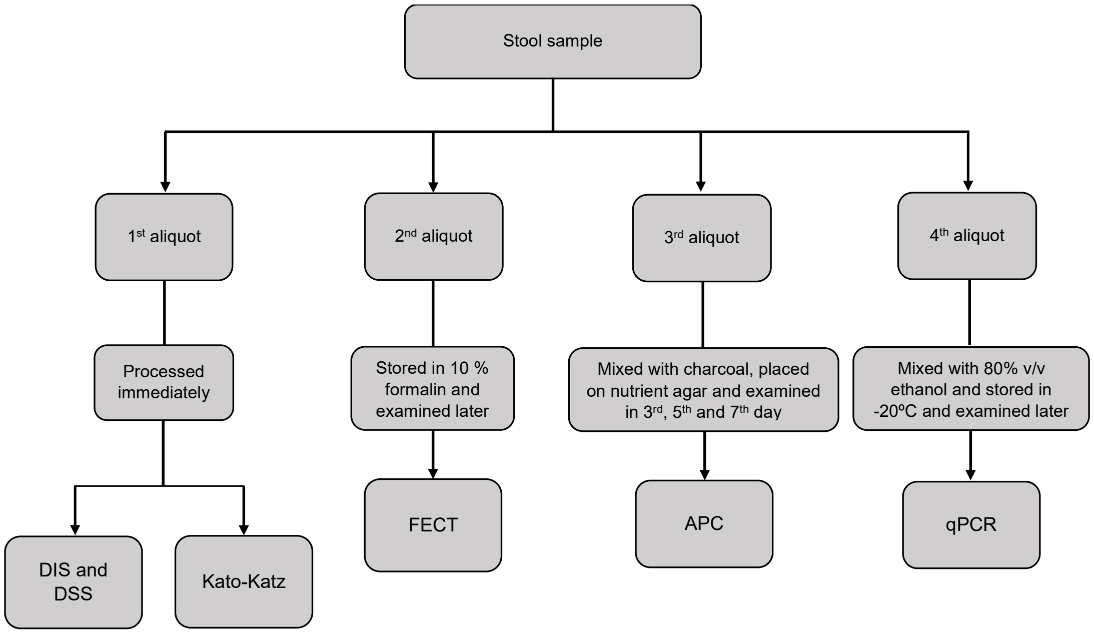

Figure. S1. The examination process of the faecal samples. DIS direct iodine smear, DSS direct saline smear, FECT formalin ether concentration technique, APC agar plate culture, qPCR polymerase chain reaction, v/v volume to volume.

A 2. Supplementary results

Table S1. Details of the number of students recruited and samples received

Educational zone	School code	Students recruited (n)	Stool samples received (n)	Stool samples received (%)	Scotch Tape samples received (n)	Scotch Tape samples received (%)
Anuradhapura	AA48	54	38	70.4	41	75.9
	AA91	45	19	42.2	22	48.9
	AA102	49	40	81.6	44	89.8
	AA35	49	36	73.5	43	87.8
	AA227	33	29	87.9	28	84.8
	AA243	36	19	52.8	22	61.1
	Total	266	181	68	200	75.2
Kekirawa	AKE1	71	38	53.5	65	91.5

	AKE113	37	31	83.8	33	89.2
	AK389	39	23	59	23	59
	AKE439	60	50	83.3	53	88.3
	Total	207	104	50.2	174	84.1
Kebithigollewa	AKB29	68	38	55.9	45	66.2
	AKB63	47	25	53.2	26	55.3
	AKB133	61	52	85.2	59	96.7
	Total	176	115	65.3	130	73.9
Galenbindunuwewa	AG8	55	50	90.9	53	96.4
	AG55	55	45	81.8	50	90.9
	AG243	47	35	74.5	39	83
	Total	157	130	82.8	142	90.4
Thambuththegama	AT60	54	37	68.5	45	83.3
	AT30	51	42	82.4	43	84.3
	AT35	53	41	77.4	47	88.7
	Total	158	120	75.9	135	85.4

n number

Table S2. Prevalence of soil-transmitted helminths and *E. vermicularis* according to the educational zone

Educational zone	School code	Microscopic STH prevalence (%)	Molecular STH prevalence (%)	*Enterobius* prevalence (%)
Anuradhapura	AA48	39.5	13.2	29.3
	AA91	47.4	57.9	31.8
	AA102	17.5	17.5	27.3
	AA35	0	30.6	32.6
	AA227	0	10.3	32.1
	AA243	0.	10.5	13.6
	Total	17.1	21.5	28.5
Thambuththegama	AT60	21.6	5.4	15.6
	AT30	16.7	59.5	32.6
	AT35	9.8	53.7	17
	Total	15.8	40.8	21.5
Kebithigollewa	AKB29	0	7.9	17.8
	AKB63	16	8.0	23.1
	AKB133	1.9	55.8	23.7
	Total	4.3	29.6	21.5

Kekirawa	AKE1	15.8	5.3	18.5
	AKE113	29	22.6	30.3
	AK389	0	4.3	26.1
	AKE439	8	48	7.5
	Total	13.4	23.9	18.4
Galenbindunuwewa	AG8	2	68	35.8
	AG55	11.1	66.7	20.0
	AG243	0.	37.1	41.0
	Total	4.6	59.2	31.7

STH soil-transmitted helminths, AA Anuradhapura/Anuradhapura, AT Anuradhapura/Thambuththegama, AKB Anuradhapura/Kenithigollewa, AKE Anuradhapura/Kekirawa, AG Anuradhapura/ Galenbindunuwewa

Table S3. Positivity by different examination methods

Examination method	*Ascaris*	*Trichuris*	Hookworm		*Strongyloides*	*Enterobius*
			Necator	*Acylostoma*		
Scotch Tape	NA	NA	NA		NA	146
Formalin ether concentration method	9	6	1		3	6
Kato-Katz	16	5	0		0	4
Agar plate culture	0	0	0		20	1
Direct saline smear	3	0	0		0	0
Direct iodine smear	3	3	1		0	1
Real-time polymerase chain reaction	98	34	35	8	101	NA
Cumulative	113	40	43		120	153

NA not applicable

Table S4. Sensitivity and specificity of the diagnostic methods in detecting *Ascaris* infection

Index test		Reference test (composite of all the index tests)		Sensitivity % (95% CI)	Specificity % (95% CI)
		+	-		
DSS	+	3	0	2.6 (0.6 – 7.6)	100 (99.3 – 100)
	-	110	513		
DIS	+	3	0	2.6 (0.6 – 7.6)	100 (99.3 – 100)
	-	110	513		
KK	+	16	0	14.2 (8.3 – 21.9)	100 (99.3 – 100)
	-	97	513		
FECT	+	9	0	7.9 (3.7 – 14.6)	100 (99.3 – 100)
	-	104	513		
PCR	+	98	0	86.7 (79 – 92)	100 (99.3 – 100)
		15	513		

DIS direct iodine smear, DSS direct saline smear, KK Kato-Katz, FECT formalin-ether concentration technique, PCR polymerase chain reaction, APC agar plate culture. Composite reference standard (DSS, DIS, KK, FECT, and PCR)

Table S5. Sensitivity and specificity of the diagnostic methods in detecting *Trichuris* infection

Index test		Reference test (composite of all the index tests)		Sensitivity % (95% CI)	Specificity % (95% CI)
		+	-		
DSS	+	0	0	0 (0 – 8.8)	100 (99.4 – 100)
	-	40	586		
DIS	+	3	0	7.5 (1.6 – 20.4)	100 (99.4 – 100)
	-	37	586		
KK	+	5	0	12.5 (4.2 – 26.8)	100 (99.4 – 100)
	-	35	586		
FECT	+	6	0	15 (5.7 – 29.8)	100 (99.4 – 100)
	-	34	586		
PCR	+	34	0	85 (70.2– 94.3)	100 (99.4 – 100)
	-	6	586		

DIS direct iodine smear, DSS direct saline smear, KK Kato-Katz, FECT formalin-ether concentration technique, PCR polymerase chain reaction, APC agar plate culture. Composite reference standard (DSS, DIS, KK, FECT, and PCR)

Table S6. Sensitivity and specificity of the diagnostic methods in detecting hookworm infection

Index test		Reference test (composite of all the index tests)		Sensitivity % (95% CI)	Specificity % (95% CI)
		+	-		
DSS	+	0	0	0 (0 – 8.2)	100 (99.4 – 100)
	-	44	582		
DIS	+	1		2.3 (0 – 12)	100 (99.4 – 100)
	-	43	582		
KK	+	0	0	0 (0 – 8.2)	100 (99.4 – 100)
	-	44	582		
FECT	+	1	0	2.3 (0 – 12)	100 (99.4 – 100)
	-	43	582		
PCR	+	43	0	97.7 (88– 99.9)	100 (99.4 – 100)
	-	1	582		

DIS direct iodine smear, DSS direct saline smear, KK Kato-Katz, FECT formalin-ether concentration technique, PCR polymerase chain reaction, APC agar plate culture. Composite reference standard (DSS, DIS, KK, FECT, and PCR)

Table S7. Sensitivity and specificity of the diagnostic methods in detecting *Strongyloides* infection

Index test		Reference test (Composite of APC+ FECT+ PCR)		Sensitivity % (95% CI)	Specificity % (95% CI)
		+	-		
APC	+	20	0	16.4 (10.3 – 24.2)	100 (99.3 – 100)
	-	102	504		
FECT	+	3	0	2.5 (0.5 – 7)	100 (99.3 – 100)
	-	119	504		
PCR	+	103	0	84.4 (76.7 – 90.4)	100 (99.3 – 100)
	-	19	504		

DIS direct iodine smear, DSS direct saline smear, KK Kato-Katz, FECT formalin-ether concentration technique, PCR polymerase chain reaction, APC agar plate culture. Composite reference standard (FECT, APC and PCR)

Table S8 Sensitivity and specificity of the diagnostic methods in detecting *Enterobius* infection

**Index test**		**Composite reference standard**		**Sensitivity %**	**Specificity%**
		**Positive**	**Negative**		
DSS	+	0	0	0 (0 – 2.2)	100 (99.3 – 100)
	-	165	523		
DIS	+	1	0	0.6 (0 – 3.3)	100 (99.3 – 100)
	-	164	523		
KK	+	4	0	2.4 (0.7 – 6.1)	100 (99.3 – 100)
	-	161	523		
FECT	+	7	0	4.2 (1.7 – 8.5)	100 (99.3 – 100)
	-	165	523		
ST	+	161	0	97.6 (93.9 – 99.3)	100 (99.3 – 100)
	-	4	523		
APC	+	1	0	0.61 (0 – 3.3)	100 (99.3 – 100)
	-	164	523		

DIS direct iodine smear, DSS direct saline smear, KK Kato-Katz, FECT formalin ether concentration technique, APC agar plate culture, ST Scotch tape. Composite reference standard (DSS, DIS, KK, FECT, and ST)
